# Orai1 calcium channels in the vasculature

**DOI:** 10.1007/s00424-012-1090-2

**Published:** 2012-03-09

**Authors:** David J Beech

**Affiliations:** 1 Multidisciplinary Cardiovascular Research Centre, University of Leeds, Leeds, LS2 9JT UK; 2 Faculty of Biological Sciences, University of Leeds, Garstang Building, Mount Preston Street, Leeds, LS2 9JT England UK

**Keywords:** Calcium channel, Blood vessel, Vascular smooth muscle cell, Endothelial cell

## Abstract

Orai1 was discovered in T cells as a calcium-selective channel that is activated by store depletion. Recent studies suggest that it is expressed and functionally important also in blood vessels, not only because haematopoietic cells can incorporate in the vascular wall but also because Orai1 is expressed and functional in vascular smooth muscle cells and endothelial cells. This article summarises the arising observations in this new area of vascular research and debates underlying issues and challenges for future investigations. The primary focus is on vascular smooth muscle cells and endothelial cells. Specific topics include Orai1 expression; Orai1 roles in store-operated calcium entry and ionic currents of store-depleted cells; blockade of Orai1-related signals by Synta 66 and other pharmacology; activation or regulation of Orai1-related signals by physiological substances and compartments; stromal interaction molecules and the relationship of Orai1 to other ion channels, transporters and pumps; transient receptor potential canonical channels and their contribution to store-operated calcium entry; roles of Orai1 in vascular tone, remodelling, thrombus formation and inflammation; and Orai2 and Orai3. Overall, the observations suggest the existence of an additional, previously unrecognised, calcium channel of the vascular wall that is functionally important particularly in remodelling but probably also in certain vasoconstrictor contexts.

## Introduction

In 2006, seminal work identified membrane proteins that were named Orais, after the Greek keepers of Heaven’s gate [20, 32, 33, 75, 76, 97, 108, 109]. An alternative name is CRACM but this is less commonly used. The proteins emerged through a study of severe combined immune deficiency (SCID), which is caused by a defect in Ca^2+^ entry of T cells [32]. A mutation in the Orai1 gene underlies this defect [33].

In predicted structure, the Orais resemble members of the extensively studied tetraspanin proteins. The Orais do not resemble other known ion pore-forming subunits of ion channels, although similarity to the regulatory (non-pore-forming) γ-subunit of voltage-gated Ca^2+^ channels has been suggested [99]. Nevertheless, several studies now indicate that Orais cluster together to form a Ca^2+^ selectivity filter and thus can be considered to be bona fide Ca^2+^ channels [108, 109]. Other tetraspanins or tetraspanin-like proteins are not known to form Ca^2+^ channels, although MS4A12 (a sequence homologue of CD20) is a candidate [53]. At the present time, there are no crystal structures for Orais, but they are suggested to have four membrane spanning segments, two extracellular loops and intracellular amino and carboxy termini [66, 109]. A pear-drop structure about 15 nm in height and 9.5 nm in width is indicated by electron microscopy [66]. Residency in the plasma membrane occurs but localisation to other compartments is not excluded. The Orais have molecular masses of about 30 kDa and these may be substantially increased by glycosylation.

Against the immunological backdrop of Orai1’s discovery, it was initially surprising that Orai1 is widely expressed but many studies now suggest expression of Orai1 not only in cells of the haematopoietic lineage [32] but also in other cell types that include vascular smooth muscle and endothelial cells (see below). The observations have started to provide important new insight into the Ca^2+^-handling capabilities of these cell types and shed light on the enigmatic process of store-operated Ca^2+^ entry (SOCE), which was first suggested in vascular smooth muscle 31 years ago [21]. Orai2 and Orai3 may also be relevant to blood vessels but available information on them is limited (see below).

This review summarises and debates evidence that Orais are important in blood vessels, with particular focus on two primary cell types of the vascular wall: vascular smooth muscle cells and endothelial cells in either their quiescent phenotypes or proliferating and migrating phenotypes. The quiescent phenotypes are especially relevant to the control of contractile tone and its regulation by endothelial factors, impacting on whole body phenomena such as peripheral resistance and tissue perfusion. The proliferating and migrating phenotypes are especially relevant to vascular development and the remodelling events of physiology and pathology that include neointimal hyperplasia, angiogenesis and endothelial repair.

## Expression of Orai1 mRNA and protein

Most of the RT-PCR, western blotting and immunocytochemical evidence for expression of Orai1 in vascular cells has arisen from studies of cultured vascular smooth muscle cells, which are migrating and proliferating but not contractile. Orai1 mRNA and protein were demonstrated in this type of cell derived from human aorta or saphenous vein [8, 13, 59], rat aorta [15, 77], rat coronary artery [29] or mouse pulmonary artery [70]. Orai1 was also detected in the A10 cell line [24], which is a model system for proliferating vascular smooth muscle cells. Orai1 protein was found to be almost undetectable in human aorta homogenate [13] or freshly isolated rat aorta vascular smooth muscle cells [77]. Orai1 protein was, however, detected in pig coronary artery [31] and rat carotid artery [107], and weak staining was reported in the smooth muscle cells of arterial sections [15, 107]. Orai1 protein was detected in rat coronary artery that had been organ-cultured for 48 h [29]. In vivo injury of arteries by physical or metabolic insult enabled clear detection of endogenous Orai1 in vascular smooth muscle cells of intact arteries [15, 31, 107]. Furthermore, a 24-h treatment of cultured vascular smooth muscle cells with platelet-derived growth factor (PDGF) led to enhanced Orai1 protein expression [72]. Therefore, the available evidence suggests relatively low Orai1 expression in native contractile vascular smooth muscle cells and higher expression in proliferating and migrating vascular smooth muscle cells, whether the phenotype is induced in vitro or in vivo. There is less RT-PCR or biochemical evidence for expression of Orai1 in endothelial cells. Nevertheless, Orai1 mRNA and protein were detected in human umbilical vein endothelial cells (HUVECs) [1, 57, 88], the HUVEC adenocarcinoma EA.hy926 cell line [6], human lung microvessel endothelial cells [88], rat pulmonary microvascular endothelial cells [81] and immortalised mouse lung endothelial cells [88]. Orai1 mRNA was also detected in endothelial colony-forming cells [80].

## Positive role of Orai1 in SOCE

A common experimental protocol applied to isolated cells is the short-term depletion of intracellular Ca^2+^ stores in the absence of extracellular Ca^2+^, for example through application of physiological agonists that cause IP_3_-induced Ca^2+^ release or application of pharmacological substances that inhibit sarcoplasmic/endoplasmic reticulum Ca^2+^ ATPase (SERCA, the pump mechanism that normally loads Ca^2+^ into the stores). Extracellular Ca^2+^ is then added back to observe Ca^2+^ entry, which is detected by an intracellular Ca^2+^ indicator. The detected rise in intracellular Ca^2+^ is often called the Ca^2+^ add-back response. The response is considerably larger in cells that have undergone store depletion, and it is primarily this observation that has led to the suggestion that store depletion triggers the opening or insertion of additional Ca^2+^ entry channels in the plasma membrane. The additional Ca^2+^ entry is often referred to as SOCE (or capacitative Ca^2+^ entry) and the channels as store-operated channels (SOCs) [95]. The experimental protocol is simple and the SOCE is striking but the complexities of the underlying biology are considerable, not least because such store depletion evokes radical changes in intracellular Ca^2+^ handling and store depletion itself is one of the classical triggers for endoplasmic reticulum (ER) stress and the associated unfolded protein response [27]. Nevertheless, studies of SOCE have yielded critical understanding of mechanisms controlling Ca^2+^ in a wide variety of cell types. Orai1 is an important element.

In cultured vascular smooth muscle cells and endothelial cells, there is SOCE. Inhibition of Orai1 expression has been found to reduce this SOCE [1, 8, 29, 57, 59, 64, 70, 77, 103]. The degree of reduction has varied from study to study but most reports agree that Orai1 plays a positive role in SOCE of these vascular cells. The studies have depended on the use of short-interfering (si) RNA [48] to suppress Orai1 expression and thus relied on the specificity of this manipulation. Nevertheless, a range of different Orai1 siRNAs have been used and the role of Orai1 has been confirmed by expression of a dominant-negative mutant of Orai1 [57, 59, 64]. Furthermore, over-expression of wild-type Orai1 has been shown to rescue SOCE after Orai1 knock-down by siRNA [59]. There have been suggestions of a critical (i.e. essential) role for Orai1 in SOCE. Evidence for such suggestions comes from studies of T cells from SCID patients or mice carrying genetic disruption of the Orai1 gene, but even in these studies residual SOCE can be observed [96]. Studies of vascular smooth muscle cells and endothelial cells in the complete absence of Orai1 have yet to be reported. Studies of cells from gene-disrupted Orai1−/− mice are complicated by immune deficiency and perinatal lethality [47].

A study of immortalised mouse endothelial cells found no effect on SOCE of Orai1 siRNA or over-expression of wild-type Orai1 or dominant-negative mutant Orai1 [88]. In human lung microvessel endothelial cells, Orai1 siRNA appeared to reduce the initial peak SOCE but a statistically significant effect was not identified [88]. The investigators suggested that, although Orai1 is expressed, it does not contribute to SOCE in these microvascular-derived endothelial cell types.

## Positive roles of Orai1 in ionic current of store-depleted cells

If SOCE does indeed result from net inward movement of Ca^2+^ across the plasma membrane, there must be an inward ionic current and it may be possible to detect it by whole-cell patch-clamp electrophysiology. Patch-clamp also has the ability to control the membrane potential and so minimise changes in membrane potential that complicate interpretation of results from intracellular Ca^2+^ indicator studies. Furthermore, the intracellular dialysis of cells with Ca^2+^ buffers, delivered by the patch-clamp pipette, can avoid or minimise intracellular Ca^2+^ rises that stimulate ion channels.

Patch-clamp studies of blood cells have, for many years, consistently revealed a distinctive inward ionic current under conditions that cause store depletion [75]. The current is referred to as calcium-release-activated Ca^2+^ (CRAC) current, or I-CRAC, and is quite well established as an electrophysiological correlate of SOCE. It is characterised by its Ca^2+^ selectivity, inward rectification and very small amplitude (a few picoamperes). Single channel currents are calculated to be well below the resolving power of patch-clamp technology. Orai1 clearly plays a major role in I-CRAC and is considered to arrange as a tetramer to form the ion pore of the underlying Ca^2+^ channels [66, 109]. It is important to note that the experimental conditions for recording I-CRAC are largely standardised and non-physiological [1, 14]. Some of these conditions have been necessary to distinguish the current from other signals. Features of the conditions include the high concentration of extracellular Ca^2+^ (usually 10 or 20 mM) and hyper-tonicity of the extracellular medium. A Na^+^-mediated ‘I-CRAC’ is often recorded in the complete absence of extracellular Ca^2+^ (divalent cation free, DVF, medium). Another common condition is a high concentration of Ca^2+^ buffer in the intracellular (patch pipette) solution (e.g. 20 mM BAPTA). The buffer serves the purposes of depleting the stores and suppressing cytosolic Ca^2+^ rises but it also lowers the basal cytosolic Ca^2+^ concentration, indiscriminately inactivating Ca^2+^-dependent processes. It is less common that I-CRAC is shown to be activated by a SERCA inhibitor when intracellular Ca^2+^ is buffered at the physiological concentration of about 100 nM.

There has been more difficulty recording I-CRAC or I-CRAC-like signals from vascular smooth muscle cells or endothelial cells [1, 37, 40, 57, 77, 98]. All of these recordings have been made from cell lines or low passage cells after primary culture. Therefore, the cells have been in proliferating and migrating phenotypes. The first report showing an I-CRAC-like signal was based on HUVECs [1]. The current amplitude was about five times smaller than that of immune cells, which is close to the resolving power of whole-cell patch-clamp. It was most convincingly shown in DVF medium and using 20 mM BAPTA in the patch pipette. It exhibited characteristics similar to those of the Na^+^ ‘I-CRAC’ of blood cells. It was diminished by Orai1 siRNA. Similar recordings were made from A7r5 and cultured rat aorta vascular smooth muscle cells [77, 98]. Similar reduction by Orai1 siRNA was observed [77].

Although investigation of the relationship to Orai1 was not shown, patch-clamp studies to seek out and determine the properties of I-CRAC were reported also in studies of EA.hy926 cells [40]. Perforated patch whole-cell recording was used in order to minimise the modification of the intracellular milieu. I-CRAC-like current was detected in response to SERCA inhibition in the presence of extracellular 10 mM Ba^2+^ and 2 mM Ca^2+^, or 0.1 mM Ba^2+^ and 10 mM Ca^2+^. The current was inwardly rectifying and small but showed a reversal potential near −11 mV [40]. Such a reversal potential, compared with the positive value described for I-CRAC in blood cells, led the authors to suggest that the current had less Ca^2+^ selectivity than I-CRAC of blood cells.

I-CRAC is not the only ionic current activated by store depletion. Various studies of proliferating or contractile vascular smooth muscle cells or endothelial cells have shown a non-selective cationic current [12, 31, 60, 63, 64, 79, 89, 94, 101, 103]. The characteristics of currents vary between studies and standardised recording conditions have not been used but the current–voltage relationship (*I*–*V*) tends to be relatively linear, the reversal potential close to or approaching 0 mV, and current observed with or without strong buffering of intracellular Ca^2+^. A recent report showed that Orai1 siRNA strongly suppressed the current in mouse aorta smooth muscle cells [103]. There is a similar current in proliferating human saphenous vein vascular smooth muscle cells [60] and it too is suppression by Orai1 siRNA [58]. The current is hard to reconcile with the properties of Orai1 Ca^2+^ channels as defined by I-CRAC. The phenomenon remains an on-going matter of investigation but, in part, it is explained by transient receptor potential (TRP) canonical channels (see below). Apparently similar non-selective cationic currents evoked by store depletion have been reported in blood cells and skeletal muscle [86, 87]. Studies of EA.hy926 cells have emphasised the complication that can arise from Na^+^–Ca^2+^ exchanger current [40] but this is not the explanation for the non-selective cationic current.

## Blockade of Orai1-related signals by Synta 66 and other pharmacology

An intriguing pharmacological agent in relation to Orai1 Ca^2+^ channels, SOCE and I-CRAC is the chemical that is referred to as Synta 66 (3-fluoropyridine-4-carboxylic acid (2′,5′-dimethoxybiphenyl-4-yl)amide). In addition to patent information (WO 2005/009954), the effects of Synta 66 on SOCE and I-CRAC in basophilic leukaemia and Jurkat T cells have been reported [26, 71]. Synta 66 does not affect plasma membrane Ca^2+^ ATPase, inward rectifier K^+^ current, hippocampal synaptic transmission, or radio-ligand binding to a range of receptors and ion channels including the L-type Ca^2+^ channel [26, 71].

Synta 66 is also an inhibitor of SOCE in proliferating vascular smooth muscle cells and endothelial cells [57, 59]. Strikingly, the IC_50_ is ~25 nM which is about two orders of magnitude better than for blood cells [57, 59]. The speed of block is relatively rapid (occurring in less than 10 s). Selectivity of Synta 66 was also suggested because there was no effect of 5 μM Synta 66 on Ca^2+^ release evoked by ATP or growth factors [57, 59]. There was no effect on Ca^2+^ entry through transient receptor potential canonical (TRPC1, 5, 6) or vanilloid (TRPV4) channels, each activated without store depletion. There was also no effect on contraction of aorta evoked by an α_1_-adrenoceptor agonist, suggesting no effect of Synta 66 on the array of systems underlying this complex contractile phenomenon [59]. There was no effect on cell viability [57]. Synta 66 appears, therefore, to be an easy-to-use, selective and potent inhibitor of SOCE. It is not currently available from a commercial supplier but may be obtained as a gift from a pharmaceutical company or synthesised relatively easily by a medicinal chemist.

The mechanism of action of Synta 66 on SOCE is unknown. It may act as an ion pore blocker of Orai1 Ca^2+^ channels, perhaps by binding directly to Orai1. However, evidence to support such a hypothesis is not reported. Indeed, the common roles of Orai1 in SOCE of vascular smooth muscle cells, endothelial cells and blood cells, set against the differential potencies of Synta 66 in these cell types (as text above), argue against Orai1 as the target protein. Nevertheless, if Orai1 is not the target protein, we do not yet have another clear candidate as the target. It is conceivable that it acts in some way to modify the coupling between depleted stores and Orai1 channels but this is not exhibited as an effect on the clustering of the store Ca^2+^ sensor [59]. It is also not exhibited as inhibition of non-selective cationic current evoked by store depletion [59].

Other inhibitors of vascular SOCE include 2-aminoethoxydiphenyl borate (2-APB), 4-methyl-4′-[3,5-bis(trifluoromethyl)-1*H*-pyrazol-1-yl]-1,2,3-thiadiazole-5-carboxanilide (BTP-2, Pyr2, or YM-58483) and lanthanides [35, 67, 77, 80]. These agents are also commonly described as TRPC channel inhibitors. Additional inhibitors of SOCE are listed by Flemming et al. [35] or include diethylstilbestrol [105], non-steroidal anti-inflammatory drugs [67], and 5-nitro-2-(3-phenylpropylamino)-benzoic acid [37]. It is not clear if any of these agents act directly as channel blockers and there is evidence that some of the agents, such as non-steroidal anti-inflammatory drugs, act indirectly [67].

The greater potency of Synta 66 on SOCE of vascular smooth muscle and endothelial cells (compared with blood cells) suggests a difference in the molecular mechanism of SOCE in these cells, even though the SOCE in all cases shows partial or strong dependence on Orai1. Different sensitivity to 2-APB [77] adds weight to this suggestion. Weaker Ca^2+^ selectivity of I-CRAC in endothelial cells has also been suggested to indicate a different molecular basis [40]. The identity of such distinction is not yet reported.

## Activation or regulation of Orai1-related signals by physiological substances and compartments

The studies described above refer to Ca^2+^ entry evoked by non-physiological stimuli. This is not to infer that they lack physiological relevance but it is necessary to consider if or when physiological stimuli can activate them. This is especially important because store depletion is a signal that leads to cell apoptosis and because physiological agonists can evoke Ca^2+^ release without causing significant store depletion, as demonstrated, for example, by simultaneous measurements of cytosolic and ER Ca^2+^ in endothelial cell lines [40, 65]. However, many investigators have applied physiological agonists to cells in the absence of extracellular Ca^2+^ and then used the Ca^2+^ add-back protocol to observe Ca^2+^ entry. While this protocol reduces confusion between Ca^2+^ release and Ca^2+^ entry, it is weakened by being a store depletion protocol (because the stores cannot refill after the Ca^2+^ release event). The experimental difficulty involved in avoiding inadvertent store depletion has been emphasised [40]. Consequently, there is only limited information about which physiological agonists activate Ca^2+^ entry that depends on Orai1 in the continuous presence of extracellular Ca^2+^ and without store depletion. Two substances that activate the channels in this situation are the important growth factors PDGF and vascular endothelial growth factor (VEGF) [57, 59]. ATP activates Synta 66-sensitive Ca^2+^ entry in the continuous presence of extracellular Ca^2+^ but it was not reported if this effect was inhibited by Orai1 siRNA [59]. Strikingly, Ca^2+^ entry stimulated by lysophosphatidylcholine (0.3 μM) was suppressed by Orai1 siRNA even though the lysophosphatidylcholine did not evoke Ca^2+^ release, suggesting Ca^2+^-release-independent activation of Orai1 channels in vascular smooth muscle cells [29].

Intriguing stimulation of SOCE-like Ca^2+^ entry by sphingosine-1-phosphate has been described in vascular smooth muscle cells [50]. While sphingosine-1-phosphate evoked Ca^2+^ release via G protein-coupled receptors, the SOCE-like signal occurred independently of sphingosine-1-phosphate receptors and was mimicked by intracellular sphingosine-1-phosphate [50]. The SOCE-like signal was not, however, shown to be Orai1-dependent.

Localisation of Orai1 to membrane density fractions containing caveolin-1 was described in studies of pulmonary microvascular endothelial cells, suggesting compartmentalisation of Orai1-dependent Ca^2+^ signalling [81]. The fractions also contained the Ca^2+^-regulated adenylyl cyclase 6. A submembrane compartment for regulation of filamin A by Ca^2+^ and cyclic AMP was suggested to play a role in the control of endothelial cell shape [81].

## Stromal interaction molecules (STIMs) and the relationship of Orai1 to other ion channels, transporters and pumps

A year before the discovery of Orai1 came the discovery of the relevance of stromal interaction molecules 1 and 2 (STIM1 and STIM2) to SOCE [20, 78]. STIMs are single-pass membrane-spanning proteins that are larger than Orais (STIM1 has a predicted mass of 75 kDa). Unlike Orais, STIMs were initially identified independently of the Ca^2+^ signalling field as glycosylated phosphoproteins located to the cell surface. Although subsequent studies confirmed STIM1 localisation in the plasma membrane, its relevance to SOCE is now most commonly described in terms of STIM1 as a protein of the ER membrane [20]. Prior to store depletion, STIM1is thought to directly bind the microtubule-plus-end-tracking protein EB1, forming comet-like accumulations [44]. STIM1 is considered to be the primary sensor of the Ca^2+^ content of the ER (or sarcoplasmic reticulum), detecting store depletion and relaying this information to Orai1 channels in the surface membrane through a complex process of conformational change, redistribution, clustering and physical association with Orai1 that clusters Orai1 proteins and activates them to enable Ca^2+^ entry [20]. STIM2 is also thought to play such a role but with a different Ca^2+^ sensitivity [20].

STIM1 and STIM2 are expressed in vascular smooth muscle cells and endothelial cells. Knock-down of the expression of either type of STIM suppresses SOCE, with STIM1 knock-down usually showing the greater effect [60, 62]. Detailed studies on other cell types have demonstrated Orai1 and STIM1 interaction [76, 104]. Less detailed studies have provided data in support of the interaction and co-localisation of endogenous Orai1 and STIM1 in vascular smooth muscle cells [17, 70]. Investigation of endogenous STIM1 has also revealed constitutive localisation to the plasma membrane and surface exposure of the amino terminus in vascular smooth muscle cells [60]. This surface STIM1 localisation is not altered by store depletion [60]. Although ER localisation of STIM1 has been suggested in vascular smooth muscle cells [92], it was not definitely identified as ER STIM1 and extensive investigation of this STIM1 has yet to be reported. A challenge with studies of STIM1 localisation is that protein tags attached to the STIM1 molecule prevent its surface localisation, tending to favour ER localisation of the STIM1 [49].

STIM1 does not only interact with Orai1 channels, it also binds ion channels that include TRPC1 and the voltage-gated Ca^2+^ channel subunit Ca_V_1.2 [98, 106]. Biochemical interaction of endogenous STIM1 and TRPC1 has been demonstrated in vascular smooth muscle cells, but there is only partial co-localisation of the proteins, suggesting that they share a function but also have discrete functions [60]. Studies in over-expression systems have suggested that STIM1 activates TRPC1 channels following store depletion [106]. In endothelial cells, store depletion evoked forward trafficking of TRPC1 and TRPV4 to the surface membrane and this effect was partially inhibited by STIM1 siRNA [63, 64]. Studies in the A7r5 vascular smooth muscle cell line have suggested that STIM1 interaction inhibits Ca_V_1.2, leading to suppression of voltage-dependent Ca^2+^ entry in store-depleted cells and a reciprocal relationship with SOCE [98]. Clustering of STIM1 and Orai1 with Na^+^–Ca^2+^ exchanger, plasma membrane Ca^2+^ ATPase and SERCA is suggested from studies of other cell types, indicating that STIM1 has a broad coordinating role for Ca^2+^ transport mechanisms and emphasising that its function is not restricted to Orai1 interaction [54]. Specifically in proliferating vascular smooth muscle cells, co-localisation of Orai1 and Na^+^–Ca^2+^ exchanger (NCX1) was observed and Orai1 siRNA suppressed NCX1 expression as well as Orai1 expression [8]. Plasma membrane Ca^2+^ ATPase (PMCA1) expression was also suppressed. Na^+^–Ca^2+^ exchange did not, however, influence SOCE [8]. SERCA2a, but not SERCA2b, expression was found to inhibit interaction between Orai1 and STIM1 [17]. Because SERCA2a was down-regulated when vascular smooth muscle cells switched to the proliferating phenotype, it was suggested that loss of this inhibitory effect of SERCA2a may contribute to the explanation for the large SOCE in proliferating vascular smooth muscle cells and the associated translocation of nuclear factor of activated T cells to the nucleus [17].

## TRPC channels and SOCE

It is suggested by the above text that Orai1 Ca^2+^ channels contribute to SOCE in vascular smooth muscle cells and endothelial cells. There are, however, also reports suggesting that TRPC channels contribute to SOCE in these cells [19, 36, 55, 60, 63, 64, 69, 82, 88–91, 93, 100, 101]. Although crystal structures are lacking for Orai and TRPC channels, there are clear suggestions that Orai and TRPC proteins are structurally unrelated. Moreover, the Orais mostly generate small Ca^2+^-selective and inwardly rectifying channels, whereas TRPC channels generate larger mixed cationic Ca^2+^- and Na^+^-permeable channels with complex rectification that includes substantial outward current. These two channel types are unlikely to participate in generating a common ion pore (i.e. they are almost certainly distinct ion channels). Furthermore, while Orai1 channels have mostly been associated with activation by store depletion, there is plenty of evidence that TRPC channels do not require store depletion in order to be activated and may often be activated even without concomitant Ca^2+^ release [2, 3, 10, 102]. The TRPC channels are not addressed in detail here, but a brief discussion of the relation to SOCE is included because the suggestion that they also contribute to SOCE is controversial, because the physiological significance of SOCE should be addressed, and because there is indication of an intimate relationship between Orai and TRPC channels, which remains poorly understood.

It may be that both Orai1 and TRPC channels contribute to vascular SOCE. This would explain why some studies show partial suppression of SOCE by Orai1 or TRPC siRNA [59, 60]. Different (apparently conflicting) results from different research groups [1, 46, 64, 88, 91] may be explained by variable relative contributions of Orai1 and TRPC depending on the exact type of cell, the condition of the cells, the culture medium, the substrate, the precise details of the experimental protocol, etc. Shared contribution to SOCE would be consistent with the substantial evidence that both types of channel interact with STIM1 [76, 106] and that STIM1 redistribution in response to store depletion has major implications for a range of Ca^2+^ transport proteins [54]. One study of cultured vascular smooth muscle cells suggested that Orai1 determines the first (transient) phase of SOCE, and TRPC1 the sustained SOCE [69, 70]. A study of EA.hy926 cells suggested a time-independent and distinct TRPC3 component of SOCE that depended on phospholipase C activity [6]. These studies suggest two distinct channels of SOCE (Orai and TRPC), but a study of HUVECs has suggested overlap and a shared Orai–TRP channel arrangement [64].

Important in this discussion is the definition of SOCE which, in practice, is a Ca^2+^ entry phenomenon observed under non-physiological conditions (see above). From a biological perspective, however, many investigators have been using SOCE as a means to understand the physiological mechanism by which stores refill following IP_3_-evoked Ca^2+^ release. The refilling process is expected to be triggered by the SOCE protocol but the protocol also has other consequences that probably do not occur, or are less prominent, when a physiological agonist evokes Ca^2+^ release under physiological conditions at a physiological concentration. One of these consequences is ER stress. Given the emerging evidence of TRPC activation by stress factors [3, 10, 28, 68], it can be anticipated that TRPC activity may be increased as a result of the SOCE (ER stress?) protocol. Potentially, dependence of SOCE on Ca^2+^-independent phospholipase A_2_ [29, 85, 103] reflects such a stress relationship because activation of this phospholipase is one of the factors involved in TRPC channel activation [4], Orai1 activation [29] and the ER stress response [56].

Another method for investigating the physiological refilling process has been the I-CRAC protocol. In many studies, however, this too is non-physiological (see above). Furthermore, the protocol is designed to isolate and highlight I-CRAC. It is quite possible that the intricate Ca^2+^ and Ca^2+^ sensor dependencies of TRPC channels [16, 51, 74, 82, 83] lead them to be suppressed or otherwise modified by the I-CRAC recording protocol, which may explain why there has been little or no resemblance of I-CRAC to ionic currents generated by over-expressed TRPC channels. Intriguingly, however, a study of freshly isolated contractile vascular smooth muscle cells showed a relatively linear *I*–*V* in I-CRAC recording conditions and strong dependence on TRPC1 [82].

In summary, it is suggested that (1) Orai1 and TRPC form distinct ion channels that do not heteromultimerise with each other; (2) Orai1 and TRPC can both contribute to the SOCE phenomenon in vascular smooth muscle cells or endothelial cells; (3) Orai1 and TRPC interact physically with STIM1 and interplay with other Ca^2+^-handling proteins such as Na^+^–Ca^2+^ exchanger; (4) Orai1 is the molecular basis of the I-CRAC Ca^2+^-selectivity filter and TRPCs do not contribute to it; (5) I-CRAC is not the only ionic current activated by store depletion; and (6) TRPCs or Orais can both be activated independently of store depletion or Ca^2+^ release.

Elucidation of the physiological mechanism by which stores refill following IP_3_-evoked Ca^2+^ release is one of the goals of the research. What we do know is that the Ca^2+^-ATPases of the stores, and especially SERCAs, are the refilling mechanism at the level of the stores and that they refill the stores using free Ca^2+^ from the cytosol. Therefore, in principle, any Ca^2+^ entry channel that contributes to the cytosolic free Ca^2+^ concentration near SERCA can contribute to store refilling; even Na^+^ entry acting indirectly via Na^+^–Ca^2+^ exchange can contribute. There is evidence that several types of Ca^2+^ entry channel can contribute in this way. The fascination in the field, however, has been that there might be a particular type of Ca^2+^ entry channel that is particularly specialised for providing Ca^2+^ to SERCA and in a restricted subcellular compartment. This specialised channel would seem to be the I-CRAC channel (i.e. the Orai1 channel). Evidence is pointing to the conclusion that such a specialised channel is a core feature across many cell types, including vascular smooth muscle cells and endothelial cells. Indeed, the original pioneering study of store refilling in vascular smooth muscle argued for a privileged Ca^2+^ entry mechanism that directly fills the stores from the extracellular medium with minimal impact on the global cytosolic Ca^2+^ concentration [21]. Nevertheless, it does not follow that this privileged mechanism is the only Ca^2+^ entry mechanism providing extracellular Ca^2+^ for store refilling or that it is the only Ca^2+^ entry channel activated by store depletion. It seems unlikely that cells would have evolved dependence on a single mechanism for store refilling when store depletion is a critical event leading to apoptosis.

## Orai1 in vascular tone (contractile phenotype)

After a period of depletion of Ca^2+^ stores in Ca^2+^-free extracellular medium, Ca^2+^ add-back was found to cause a contractile response in aorta that was larger in stroke-prone spontaneously hypertensive rats [38]. Delivery of anti-Orai1 antibody by the Chariot technique suppressed the contraction [38]. These data suggest that functional Orai1 channels exist in contractile vascular smooth muscle cells of the aorta. Superficially, the observation conflicts with the finding that Synta 66 had no effect on α1-adrenoceptor-mediated contraction of mouse aorta [59]. The Synta 66 result is, however, consistent with the study of rat aorta which showed that SOCE inhibitors were ineffective when the Ca^2+^ add-back response was not preceded by exposure to a SERCA inhibitor in normotensive animals [38]. Therefore, the preliminary conclusion from these studies is that SOCE is not particularly important in contractile function of physiological aorta unless there is substantial store depletion. The suggestion is reminiscent of prior studies, for example on cerebral arterioles, which have also suggested that SOCE generates an intracellular Ca^2+^ elevation that is not well coupled to contraction [34]. However, investigation of rat coronary artery has shown that contractions evoked by urotensin-II, the α1-adrenoceptor agonist phenylephrine or lysophosphatidylcholine are suppressed in arterial segments cultured for 48 h after Orai1 siRNA delivery [29]. The effects were observed in the continuous presence of extracellular Ca^2+^, and therefore, they suggest that Orai1 channels are important in physiological contractile responses of this artery. A note of caution, however, is that previous work on basilar artery suggested that SOCE had no effect on contraction of freshly isolated artery but strong effect on contraction after organ culture of the artery for 72 h [11, 12]. Although vessels can remain contractile after periods of culture, early remodelling events are likely to have taken place (see below). Further studies would be valuable on the relevance of Orai1 to contractile function in various blood vessels and in relation to endothelium-dependent vasodilatation.

## Orai1 in vascular remodelling (migrating and proliferating phenotypes)

Several studies have found that expression of Orai1 mRNA and protein are up-regulated when vascular smooth muscle cells undergo their switch from the contractile to the non-contractile (migrating and proliferating) phenotype (see above). It has also been observed that SOCE is larger in proliferating vascular smooth muscle cells [41, 42] and many of the studies of SOCE and Orai1 have focused on vascular smooth muscle cells in culture, which causes rapid switching to the non-contractile phenotype. Moreover, inhibition of migration has been observed after Orai1 knock-down by siRNA, suggesting an important role of Orai1 in the non-contractile phenotype [59, 77]. An inhibitory effect of Orai1 siRNA on cell number of rat aorta vascular smooth muscle cells was reported [77], but the effect was relatively small and the number of human saphenous vein vascular smooth muscle cells was unaffected at the same 48-h time point, suggesting a preferential effect on migration [59]. In studies of human aorta vascular smooth muscle cells, there was a reduction in cell number at the later time point of 77 h [8]. Similarly, Synta 66 inhibited migration but not the number of vascular smooth muscle cells [59]. Further support for a role of Orai1 in the migrating phenotype came from the finding that Orai1 siRNA markedly inhibited the sustained elevation of intracellular Ca^2+^ evoked by PDGF in the continuous presence of extracellular Ca^2+^ [59]; this finding is important because PDGF is the primary growth factor driving smooth muscle cell recruitment during vascular development and pathological remodelling [52]. In vivo studies have found that Orai1 knock-down strongly reduces neointimal formation in carotid artery [46, 107], similar to the effect of STIM1 knock-down [7, 45]. Similarly, STIM1 knock-down suppresses vascular smooth muscle cell migration in vitro [60]. Collectively, these findings suggest that Orai1 channels and SOCE play key positive roles in enabling efficient vascular smooth muscle cell remodelling, working with a range of other ion channels that include TRPC1 and K_V_1.3 potassium channel [9, 23, 25, 55].

Endothelial cells also remodel using a phenotype that displays migrating and proliferating properties. Knock-down of Orai1 by siRNA inhibits the migration [57] and proliferation [1] of HUVECs. It also markedly inhibits the sustained elevation of intracellular Ca^2+^ evoked by VEGF [57], the primary growth factor driving endothelial cell migration and endothelial remodelling events such as angiogenesis [73]. In vitro tube formation, which mimics features of angiogenesis, was inhibited by Orai1 siRNA or dominant-negative mutant Orai1 [57]. Exogenous wild-type Orai1 rescued the tube formation after Orai1 knock-down by siRNA [57]. Synta 66 inhibited endothelial cell migration and in vitro tube formation and suppressed angiogenesis in vivo in the chick chorioallantoic membrane [57]. Similarly, suppression of STIM1 inhibited angiogenesis in vivo [22]. A study of EA.hy926 cells, by contrast, found no effect of Orai1 siRNA on in vitro endothelial tube formation, a difference the authors suggest may have been due to the absence, or low concentration of, VEGF in their studies [5]. A reduction in EA.hy926 cell proliferation by Orai1 siRNA was observed [5], similar to findings in HUVECs [1]. Proliferation and tubulogenesis of endothelial colony forming cells in the presence of VEGF was inhibited by BTP-2 [30]. Overall, the findings suggest that Orai1 channels and SOCE are important in endothelial cell proliferation, VEGF signalling, VEGF-driven endothelial cell migration and VEGF-driven angiogenesis.

## Orai1 in thrombus and inflammation

This review focuses on two dominant cell types of the vascular wall but it should be borne in mind that Orai1 is also expressed in blood cells (T cells, monocytes, platelets, etc.) which can interact with and integrate in the vascular wall as part of inflammatory and thrombotic events. Numerous studies suggest the importance of Orai1 channels in thrombus formation and inflammation [18, 32, 39].

## Orai2 and Orai3

Orai2 and Orai3 mRNAs are also detected in vascular smooth muscle cells and endothelial cells [1, 8, 59, 80], showing either substantial abundances that are greater than those of Orai1 mRNA [8, 59] or minimal abundance [88]. Orai2 and Orai3 proteins have also been detected [13, 17, 24, 77, 88]. Orai2 and Orai3 were up-regulated in proliferating compared with contractile vascular smooth muscle cells [8]. Knock-downs of Orai2, Orai3 or Orai2 and Orai3 by siRNAs have shown no effect on SOCE or basal cytosolic Ca^2+^ in proliferating vascular smooth muscle cells [8, 15, 59, 77] even though over-expression studies in the human embryonic kidney (HEK) 293 cell line have suggested that Orai2 or Orai3 is capable of reconstituting an I-CRAC [61]. There was also no effect of Orai2 or Orai3 siRNA on vascular smooth muscle cell migration or proliferation [8, 15]. Intriguing studies of an endogenous arachidonate-regulated Ca^2+^ (ARC) channel in HEK 293 cells have suggested that this channel is formed from a pentameric arrangement of Orai1 with Orai3 [84]. It is not reported if such a channel is relevant to the vasculature.

## Conclusions and future challenges

The evidence points to Orai1 as a novel Ca^2+^ channel of blood vessels. The strongest evidence for expression and roles of Orai1 in the vasculature is in remodelling events that relate to neointimal hyperplasia and angiogenesis. Orai1 can play significant positive roles in migrating and proliferating behaviours of vascular smooth muscle and endothelial cells, all of which are important in events such as neointimal hyperplasia and angiogenesis. There is less evidence for the expression and roles of Orai1 in the contractile state of blood vessels but function is indicated and may be important in specific vessels under certain conditions. In both the remodelling and contractile contexts, there is need for more information on the expression and functional relevance of endogenous Orai1 channels especially in freshly isolated cells and tissues and, in vivo, in animals under physiological and pathological conditions.

A fundamental implication from Orai1’s discovery is that it represents a long-sought, privileged and widespread mechanism for refilling of depleted Ca^2+^ stores. It would seem to be true that Orai1 provides such a mechanism, but strengths of the argument depend substantially on principles developed from studies of cell types other than vascular smooth muscle and endothelial cells or from over-expression approaches in cell lines. Reports on vascular smooth muscle cells and endothelial cells provide numerous indications that store depletion is associated with the activation or insertion not only of Orai1 channels but also additional types of Ca^2+^-permeable channel that impact on cytosolic Ca^2+^ concentrations directly or indirectly. The relationship between these channels and Orai1 requires further investigation and would benefit from the application of new technical approaches that provide better resolution in subcellular space, better information about associations between endogenous proteins in physiological cells and better information about activation of the channels in physiological and pathological contexts when Ca^2+^ signalling occurs in three-dimensional structures that are in slow turnover (quiescence) or actively remodelling.

An important step in the short term is to better address the relevance to physiological settings of experimentally induced store depletion events and the SOCE phenomenon. Several studies suggest that Ca^2+^ release is not necessarily associated with store depletion and thus that a refilling process may be activated and maintained in the absence of store depletion. The reported activation of Orai1-dependent Ca^2+^ entry by PDGF or VEGF in the continuous presence of extracellular Ca^2+^ suggests the involvement of Orai1 in store refilling even when there is little or no store depletion. If there is indeed such efficient store refilling via Orai1, it raises questions about the physiological activation mechanism of Orai1 and the appropriateness of considering Orai1 only in terms of the store depletion-activated Orai1–STIM1 I-CRAC complex. Dependence of non-selective cationic current on Orai1 [103] and the greater effect of Orai1 siRNA than Synta 66 on vascular smooth muscle cell migration [59] are suggestive of multiple rather than singular functions of Orai1. What these other functions are and whether they arise indirectly through the I-CRAC mechanism remain to be determined.

One of the most obvious problems in the field is the apparently conflicting published data sets on the molecular basis of SOCE. Put simply: Is SOCE mediated by Orai1, TRPC, other channels, etc., or all of them? How can different investigators use apparently similar experimental protocols and end up with such widely differing results and conclusions (e.g. Orai1 explains all of SOCE and TRPC none, or vice versa)? It would be helpful if experimental conditions were standardised. Another way forward would be to decrease emphasis on the SOCE phenomenon and focus attention instead on physiological activators of the channels and studies in physiological conditions. A further way forward is to accept that multiple channel types can contribute to SOCE in cells in vitro in planar culture or suspension but that the physiological relevance of these contributions depends on the exact cell type and the context. An intriguing study, for example, suggested the importance of the TRPC4 channel at the point in time when endothelial cells make contact [43]. Such a subtle but important effect would variably contribute to in vitro planar cell culture studies depending on the confluence of the cells. Also important in such a situation would be the substrate on which the cells were grown and placed during experiments.

Additional challenges ahead involve addressing (1) whether the vascular I-CRAC channel has a distinct molecular component compared with the I-CRAC channel in T cells, conferring a basis for distinction by pharmacology and, potentially, therapeutic drugs; (2) the roles of Orai2 and Orai3 in blood vessels (e.g. Is an ARC channel relevant?); and (3) the nature of the down-stream pathways of Orai1 channels and other channel types contributing to SOCE (there may be, for example, discrete consequences of activating Orai1- compared with TRPC1-containing channels [60]).

The discovery of Orai1 in T cells has led to an interesting and lively period of research in the Ca^2+^ signalling and vascular fields. A previously unrecognised channel type of vascular smooth muscle cells and endothelial cells seems to have been identified and appears to have important functional consequences that could be relevant and significant for fundamental understanding and new therapeutic strategies. We are, however, at the beginning of this period of investigation and there is much still to learn and resolve. Application of new experimental methods and emphasis on other types of existing methods will be necessary as the field progresses.
